# Excited-State Aromaticity Reversals in Naphthalene and Anthracene

**DOI:** 10.1021/acs.jpca.3c00485

**Published:** 2023-04-03

**Authors:** Peter B. Karadakov, Muntadar A. H. Al-Yassiri

**Affiliations:** Department of Chemistry, University of York, Heslington, York YO10 5DD, U.K.

## Abstract

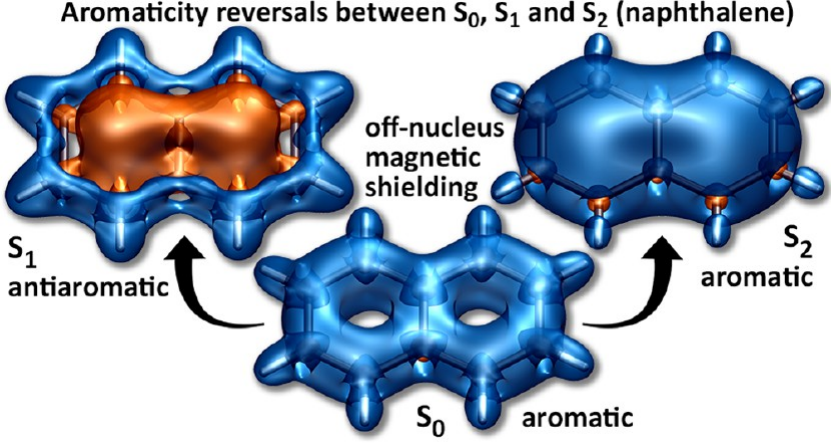

Aromaticity reversals between the electronic ground (S_0_) and low-lying singlet (S_1_, S_2_) and triplet (T_1_, T_2_, T_3_) states of naphthalene and anthracene are investigated by calculating the respective off-nucleus isotropic magnetic shielding distributions using complete-active-space self-consistent field (CASSCF) wavefunctions involving gauge-including atomic orbitals (GIAOs). The shielding distributions around the aromatic S_0_, antiaromatic S_1_ (^1^L_b_), and aromatic S_2_ (^1^L_a_) states in naphthalene are found to resemble the outcomes of fusing together the respective S_0_, S_1_, and S_2_ shielding distributions of two benzene rings. In anthracene, ^1^L_a_ is lower in energy than ^1^L_b_, and as a result, the S_1_ state becomes aromatic, and the S_2_ state becomes antiaromatic; the corresponding shielding distributions are found to resemble extensions by one ring of those around the S_2_ and S_1_ states in naphthalene. The lowest antiaromatic singlet state of either molecule is found to be significantly more antiaromatic than the respective T_1_ state, which shows that it would be incorrect to assume that the similarity between the (anti)aromaticities of the S_1_ and T_1_ states in benzene, cyclobutadiene, and cyclooctatetraene would be maintained in polycyclic aromatic hydrocarbons.

## 1 Introduction

A molecule that is aromatic or antiaromatic in its electronic ground state can experience a reversal of aromaticity upon transition to a ππ* excited electronic state; this reversal is accompanied by profound changes in its electronic structure and properties. Behavior of this type has been aptly named the molecular analogue of Robert Louis Stevenson’s “Dr Jekyll and Mr Hyde”,^1^ and it has numerous applications, which include designing molecules with light-controllable behavior, for example, molecular photoswitches,^2^ molecular motors,^3,4^ “flapping” flurorophores,^5−7^ and rationalizing experimental evidence about photochemical reactions such as excited-state intramolecular proton transfers.^8,9^ Further applications of excited-state aromaticity reversals have been discussed in a recent review.^10^

Excited-state aromaticity is usually associated with Baird’s rule,^11^ according to which the familiar Hückel 4*n* + 2 and 4*n* rules for electronic ground-state aromaticity in cyclic conjugated hydrocarbons are reversed in the lowest triplet state: rings with 4*n* π electrons switch from antiaromatic to aromatic, while those with 4*n* + 2 π electrons switch from aromatic to antiaromatic. Similar aromaticity reversals have been shown to take place in the lowest singlet excited state.^12−15^

The variations in isotropic magnetic shielding, σ_iso_(**r**) = 1/3[σ_*xx*_(**r**) + σ_*yy*_(**r**) + σ_*zz*_(**r**)], within molecular space, for the electronic ground (S_0_), the lowest triplet (T_1_), and for the first and second singlet excited states (S_1_ and S_2_) of benzene (C_6_H_6_),^15^ square cyclobutadiene (C_4_H_4_),^15^ and regular octagonal cyclooctatetraene (C_8_H_8_)^16^ indicate that bonding and levels of aromaticity in the aromatic S_1_ and T_1_ states of C_4_H_4_ and C_8_H_8_ are very similar; the same applies to bonding and levels of antiaromaticity in the antiaromatic S_1_ and T_1_ states of C_6_H_6_. These observations might prompt the assumption that, in general, the levels of reversed aromaticity in the S_1_ and T_1_ states of a molecule aromatic or antiaromatic in S_0_ should be very much the same, and that using results for the T_1_ state, which is much easier to access computationally, for example, through spin-unrestricted density functional theory (UDFT) calculations, it would be possible to predict the properties of the S_1_ state. One of the aims of this paper is to show that this assumption can be widely off the mark: the comparisons between bonding and levels of antiaromaticity in the first antiaromatic singlet excited state and T_1_ state of naphthalene (C_10_H_8_) and anthracene (C_14_H_10_) reveal profound differences.

Naphthalene and anthracene, which contain two and three ortho-fused benzene rings, respectively, are the *n* = 2 and *n* = 3 successors of benzene in the [*n*]acene series. The trends in the ground-state aromaticities of linear polyacenes have been the subject of numerous theoretical studies. According to Clar’s rule, all six-membered rings in linear polyacenes with closed-shell ground states should exhibit identical levels of local aromaticity.^17^ However, the values of several NICS (nucleus-independent chemical shift) aromaticity indices, including the popular NICS(0)^18^ and NICS(1),^19,20^ off-nucleus isotropic shieldings with a reversed sign, −σ_iso_(**r**), calculated at the ring center and 1 Å above the ring center, respectively, suggest that both the local aromaticity of the central rings in [*n*]acenes and the average aromaticity per ring, which characterizes the aromaticity of the whole molecule, are increasing with size; both rings in naphthalene, as well as the central ring in anthracene, are predicted to be more aromatic than the benzene ring;^21,22^ the central ring in anthracene is predicted to be more aromatic than the outer rings. These findings have been causing some discomfort among theoretical chemists and, so far as anthracene is concerned, have become known as the “anthracene problem”.^23,24^ Other aromaticity indices exhibit behavior similar to NICS,^25^ and this topic is continuing to attract research interest.^26,27^ Clearly, most of the existing NICS data for anthracene and higher acenes cannot be used to explain the experimental observations about the increased reactivity of the central rings. However, these observations can be easily rationalized by examining the levels of aromatic stabilization of the corresponding transition states and end products.^21^

One possibility that has not been investigated in detail so far is that the observed NICS behavior in [*n*]acenes could be due, at least in part, to the level of theory used in the calculations. The NICS values reported in refs (21, 22) were obtained with restricted DFT (RDFT) methods, combined with IGLOs (individual gauges for localized orbitals), or with GIAOs (gauge-including atomic orbitals), respectively. It has been established that the RB3LYP solutions for [*n*]acenes become nonsinglet unstable for *n* ≥ 6, and it is possible to find lower-energy spin-unrestricted B3LYP (UB3LYP) solutions, which exhibit relatively low levels of spin contamination.^28^ In fact, a closely related nonsinglet instability of the spin-restricted Hartree-Fock (RHF) wavefunctions for linear polyacenes, leading to lower-energy spin-unrestricted HF (UHF) wavefunctions, had been reported in earlier studies;^29−32^ RHF nonsinglet instabilities were observed for even smaller oligoacenes with *n* = 3 and *n* = 4, anthracene and tetracene,^32^ but the levels of spin contamination of the respective UHF wavefunctions were found to be very high. UB3LYP-GIAO NICS calculations expanding on the findings of ref (28) suggest that, in heptacene, octacene, and nonacene (*n* = 7–9), the highest levels of local aromaticity occur not at the central but at the penultimate rings at either side of the oligoacene, and aromaticity decreases toward the middle of the oligoacene; this decrease is more pronounced in longer oligoacenes.^33^ The more detailed UB3LYP-GIAO σ_iso_(**r**) contour plot for [7]acene indicates that the terminal rings feature localized double bonds with no significant aromatic character whereas the internal rings are likely to host singlet biradical pairs at the para positions.^27^

The magnetic properties of benzene and naphthalene computed using full π space complete-active-space self-consistent field (CASSCF) wavefunctions constructed from GIAOs, CASSCF(6,6)-GIAO and CASSCF(10,10)-GIAO for benzene and naphthalene, respectively,^8^ show that, according to both of NICS(0) and NICS(1), each of the six-membered rings in naphthalene is slightly less aromatic than benzene; the difference in favor of benzene is more pronounced in two other NICS indices, NICS(0)_*zz*_ and NICS(1)*_zz_,*^34,35^ which correspond to the *zz* (out-of-plane) component of shielding tensor with an inversed sign, −σ_*zz*_(**r**), calculated at the locations used for NICS(0) and NICS(1). The isotropic magnetic susceptibilities, χ_iso_, and the out-of-plane components of the magnetic susceptibility tensors, χ_*zz*_, for benzene and naphthalene, evaluated at these CASSCF-GIAO levels of theory and taken as “per ring” values, also confirm that a six-membered naphthalene ring is less aromatic than benzene. Both the UB3LYP-GIAO and CASSCF-GIAO results suggest that including nondynamic electron correlation effects when calculating NICS and other magnetic properties might help resolve the “anthracene problem”; this conjecture is investigated further in the current work.

In this paper, we use magnetic properties including NICS, magnetic susceptibilities, off-nucleus isotropic magnetic shielding isosurfaces, and contour plots, calculated using state-optimized full π space CASSCF-GIAO wavefunctions, to study aromaticity, antiaromaticity, and chemical bonding in the low-lying ππ* electronic states of naphthalene and anthracene. The electronic states examined, for both molecules, comprise the three lowest singlet and three lowest triplet electronic states, S_0_, S_1_, S_2_, T_1_, T_2_, and T_3_. The three singlet states match those of benzene and square cyclobutadiene studied in ref (15); in addition to T_1_, the triplet states now include T_2_ and T_3_. We show that the profoundly different off-nucleus isotropic shielding distributions surrounding aromatic and antiaromatic low-lying electronic states of benzene and square cyclobutadiene^15^ are also observed, with notable modifications, in some of the aromatic and antiaromatic electronic states of the two examples of polycyclic aromatic hydrocarbons (PAHs) studied in this paper. Benzene and square cyclobutadiene display consecutive aromaticity reversals between their S_0_, S_1_, and S_2_ states: benzene is aromatic in S_0_, becomes antiaromatic in S_1_, and then reverts to aromatic in S_2_; square cyclobutadiene alternates between being antiaromatic in S_0_, aromatic in S_1_, and antiaromatic in S_2_.^15^ It is reasonable to expect benzene-like aromaticity switching in the S_0_, S_1_, S_2_ sequences in naphthalene and anthracene, but there is a detail that could lead to a different pattern of aromaticity reversals in anthracene and larger linear polyacenes: in Platt’s notation,^36^ S_1_ and S_2_ in benzene and naphthalene correspond to the^1^L_b_ and ^1^L_a_ states, respectively, but in anthracene and beyond, the order is reversed, and ^1^L_a_ is lower in energy than ^1^L_b_. The aromaticity reversal between S_0_ and T_1_ in a molecule with an aromatic ground state is predicted by Baird’s rule,^11^ but the possibility of further aromaticity reversals in the sequence T_1_, T_2_, T_3_ has not been investigated previously. We show that the CASSCF-GIAO, UB3LYP-GIAO, and UHF-GIAO methods make different predictions about the levels of T_1_ antiaromaticity in naphthalene and anthracene: UB3LYP-GIAO exaggerates, and UHF-GIAO downplays T_1_ antiaromaticity in these two PAHs.

## 2 Computational Details

In all calculations on naphthalene reported in this paper, we used the *D*_2h_ gas-phase ground-state geometry established by Baba et al.^37^ through a combination of ultrahigh-resolution laser spectroscopy and *ab initio* calculations. For anthracene, we used the *D*_2h_ gas-phase ground-state geometry optimized at the MP2(Full)/cc-pVTZ level (second-order Møller-Plesset perturbation theory including all orbitals in the correlation treatment) with GAUSSIAN,^38^ under the “VeryTight” convergence criteria.

The S_0_, S_1_, S_2_, T_1_, T_2_, and T_3_ electronic states of naphthalene and anthracene were described using state-optimized full π space CASSCF(10,10) (“10 electrons in 10 orbitals”) and CASSCF(14,14) (“14 electrons in 14 orbitals”) wavefunctions. The basis sets used in the CASSCF calculations on these states were 6-311+G(d) for naphthalene and 6-311G(d) for anthracene. All CASSCF-GIAO calculations reported in this paper were carried out using the MCSCF-GIAO (multiconfigurational SCF with GIAOs) methodology introduced in refs (39) and (40) and implemented in the Dalton program package.^41^

As a consequence of the use of ground-state geometries for all of the electronic states of naphthalene and anthracene included in this study, the comparisons between the properties of these states are in the context of vertical excitations. In line with previous work on NICS^13−15,42,43^ and ring currents^44^ in triplet systems, the CASSCF-GIAO shielding data in the T_1_, T_2_, and T_3_ electronic state of naphthalene and anthracene reported in this paper include the contributions arising from the perturbation to the wavefunction only (often referred to as “orbital” contributions in single-determinant approaches). This choice is convenient for the purposes of the current study, as the values reported for a triplet state can be compared directly to those for singlet states. A more rigorous treatment would need to consider the large terms associated with the interaction between the electron spin angular momentum and magnetic field.^45,46^

State-optimized and state-averaged CASSCF calculations account only for nondynamic electron correlation effects and do not always reproduce the correct energy ordering of the electronic excited states. Examples include benzene, for which the order of the S_3_ and S_4_ states is inversed by both state-optimized CASSCF^47^ and state-averaged CASSCF;^48^ naphthalene, for which the ordering of several singlet excited states, including S_2_ and S_4_, is not reproduced correctly by state-averaged CASSCF;^48^ anthracene, for which even the ordering of S_1_ and S_2_, is inversed by state-averaged CASSCF.^49,50^ State-averaged CASSCF reproduces the correct energy ordering of the T_1_, T_2_, and T_3_ states for naphthalene,^48^ but not for anthracene, for which the order of the T_2_ and T_3_ states is reversed.^49^ Getting the excited states in the correct order requires the inclusion of dynamic electron correlation effects, for example, through CASPT2 (second-order perturbation theory with a CASSCF reference) and, in the case of anthracene, the use of a larger basis set.^50^ Given that CASPT2 theory and codes for the calculations of magnetic properties are not currently available, and assuming that aromaticity and antiaromaticity depend mainly on the π electrons, the selection of state-optimized π space CASSCF wavefunctions for the electronic states of naphthalene and anthracene examined in this paper was guided by wavefunction symmetry and dominant configurations, in preference to energies. Theory and experiment (see refs (48−50) and references therein) indicate that the S_1_ and S_2_ states of naphthalene are 1^1^B_3u_ (^1^L_b_) and 1^1^B_2u_ (^1^L_a_), respectively; the ordering of these states is reversed in anthracene, in which S_1_ and S_2_ are identified as 1^1^B_2u_ (^1^L_a_) and 1^1^B_3u_ (^1^L_b_), respectively.^50,51^ In both molecules, the ^1^L_a_ state is dominated by the HOMO → LUMO excitation, while the main contributions to the ^1^L_b_ state come from the HOMO −1 → LUMO and HOMO → LUMO +1 excitations. This information is sufficient for choosing the appropriate state-optimized CASSCF approximations to the S_1_ and S_2_ states of naphthalene and anthracene (the ordering of S_1_ and S_2_ in anthracene is still reversed in state-optimized CASSCF); for T_1_, T_2_, and T_3_, state-optimized CASSCF behaves in the same manner as state-averaged CASSCF, reproducing the correct energy ordering of these states for naphthalene, and reversing the order of T_2_ and T_3_ for anthracene.

Volume and in-plane data describing the changes in magnetic shielding for the various electronic states of naphthalene and anthracene in the regions of space surrounding the molecules were obtained by evaluating off-nucleus magnetic shielding tensors, σ(**r**), for each electronic state, at regular two- or three-dimensional grids of points with a spacing of 0.05 Å. For the electronic states of naphthalene, we used a three-dimensional grid in the shape of a 9 Å × 7 Å × 5 Å rectangular box, centered at the inversion center, aligned with the three *C*_2_ symmetry axes and extending 2.5 Å above and below the molecular plane.

Our preliminary calculations showed that, even with the smaller 6-311G(d) basis used for anthracene, the computational effort required to obtain shielding data over a three-dimensional grid of a sufficient size at the CASSCF(14,14)-GIAO level would be too high. Therefore, for the electronic states of anthracene, use was made of two 11 Å × 7 Å two-dimensional grids, a horizontal grid in the molecular plane (σ_h_), centered at the inversion center and aligned with the *C*_2_ symmetry axes in that plane, and a grid in a vertical σ_v_ symmetry plane, centered at the inversion center, perpendicular to the molecular plane and bisecting all three benzene rings. To reduce computational effort, σ(**r**) tensors were calculated only at symmetry-unique grid points and replicated by symmetry. For visualization purposes, all σ_iso_(**r**) values from the three-dimensional grid for each electronic state of naphthalene were assembled in a GAUSSIAN cube file.^52^ The data from the two-dimensional grids for the electronic states of anthracene were used to construct σ_iso_(**r**) contour plots; analogous contour plots for naphthalene were constructed by extracting the required data from the three-dimensional grids.

The levels of antiaromaticity in the T_1_ states of naphthalene and anthracene predicted by the CASSCF-GIAO, UB3LYP-GIAO, and UHF-GIAO methods were compared using contour plots constructed from UB3LYP-GIAO and UHF-GIAO shielding data at two-dimensional grids analogous to those described above and calculated using the 6-311+G(d) basis for naphthalene and the 6-311G(d) basis for anthracene. These UB3LYP-GIAO and UHF-GIAO calculations were carried out using GAUSSIAN.^38^

NICS(0), NICS(1), NICS(0)_zz_, NICS(1)_*zz*_, χ_iso_, and χ_*zz*_ values for the S_0_, S_1_, S_2_, T_1_, T_2_, and T_3_ states of naphthalene and anthracene were calculated at the levels of theory used to obtain the data required for the construction of shielding isosurfaces and contour plots.

To obtain a better understanding of the method dependence of magnetic properties associated with the “anthracene problem”, additional calculations producing ground-state NICS(0), NICS(1), NICS(0)_zz_, NICS(1)_*zz*_, χ_iso_, and χ_*zz*_ values for benzene, naphthalene, and anthracene were carried out using π space CASSCF wavefunctions, CASSCF(6,6)-GIAO, CASSCF(10,10)-GIAO, and CASSCF(14,14)-GIAO, respectively, MP2-GIAO, and two coupled-cluster approaches, CC2-GIAO and CC3-GIAO, all within the 6-311G(d) basis. For benzene, use was made of the experimental *D*_6h_ gas-phase ground-state geometry established through analysis of the ν_4_ vibration-rotation bands of C_6_H_6_ and C_6_D_6_.^53^ The MP2-GIAO, CC2-GIAO, and CC3-GIAO calculations were performed using CFOUR.^54^

## 3 Results and Discussion

The energies of the state-optimized full π space CASSCF(10,10)/6-311+G(d) and CASSCF(14,14)/6-311G(d) wavefunctions for the S_0_, S_1_, S_2_, T_1_, T_2_, and T_3_ states of naphthalene and anthracene are shown in Table 1 together with the corresponding vertical excitation energies.

**Table 1:** State-Optimized Full π Space CASSCF Energies of the S_0_, S_1_, S_2_, T_1_, T_2_, and T_3_ States of Naphthalene and Anthracene, and UHF and UB3LYP Energies of the T_1_ States (*E*_h_); CASSCF Vertical Excitation Energies Δ*E* (eV) (For Details, See Text)^a^

molecule	method	state	total energy	Δ*E*
C_10_H_8_	CASSCF	S_0_ (1^1^A_g_)	–383.544114	0.00
		S_1_ (1^1^B_3u_)	–383.388916	4.22
		S_2_ (1^1^B_2u_)	–383.310389	6.36
		T_1_ (1^3^B_2u_)	–383.433004	3.02
		T_2_ (1^3^B_3u_)	–383.385242	4.32
		T_3_ (1^3^B_1g_)	–383.381271	4.43
	UHF	T_1_ (2.1996)	–383.317073	
	UB3LYP	T_1_ (2.0205)	–385.861594	
C_14_H_10_	CASSCF	S_0_ (1^1^A_g_)	–536.259256	0.00
		S_1_ (1^1^B_2u_)	–536.065396	5.28
		S_2_ (1^1^B_3u_)	–536.121148	3.76
		T_1_ (1^3^B_2u_)	–536.175907	2.27
		T_2_ (1^3^B_3u_)	–536.115540	3.91
		T_3_ (1^3^B_1g_)	–536.122830	3.71
	UHF	T_1_ (2.1079)	–536.018487	
	UB3LYP	T_1_ (2.0182)	–539.558722	

a 6-311+G(d) and 6-311G(d) basis sets for naphthalene and anthracene, respectively; CASSCF(10,10) and CASSCF(14,14) for naphthalene and anthracene, respectively. For UHF and UB3LYP, the ⟨*Ŝ*^2^⟩ values are provided in brackets next to the T_1_ state symbol. In Platt’s notation 1^1^B_3u_ ≡ ^1^L_b_ and 1^1^B_2u_ ≡ ^1^L_a_.

The current vertical excitation energies are in good agreement with the results of other authors for naphthalene^48−50^ and anthracene,^49,50^ obtained using state-averaged π space CASSCF(10,10) and CASSCF(14,14) wavefunctions with different basis sets. In addition, Table 1 includes the energies and ⟨*Ŝ*^2^⟩ values for the UHF and UB3LYP approximations to the T_1_ states of the two molecules. The levels of spin contamination are relatively low, especially in the case of UB3LYP, and decrease with the increase of the length of the acene. Despite the use of different orbitals for different spins, the UHF T_1_ energies are significantly higher than their π space CASSCF counterparts.

**Figure 1:**
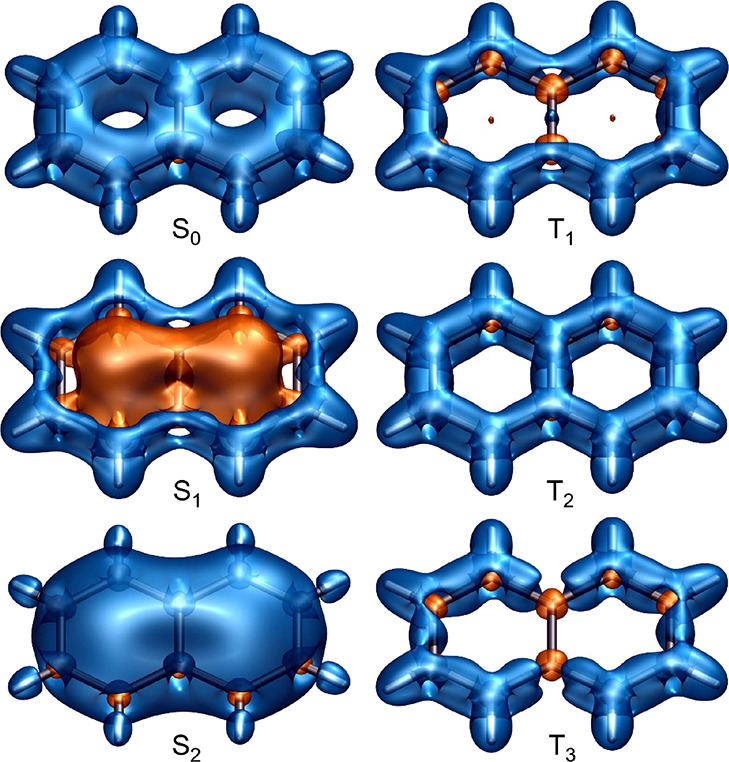
Isotropic shielding isosurfaces for the S_0_, S_1_, S_2_, T_1_, T_2_, and T_3_ states of naphthalene at σ_iso_(**r**) = ±16 ppm (positive/negative isovalues in blue/orange) [CASSCF(10,10)-GIAO/6-311+G(d) results].

**Figure 2:**
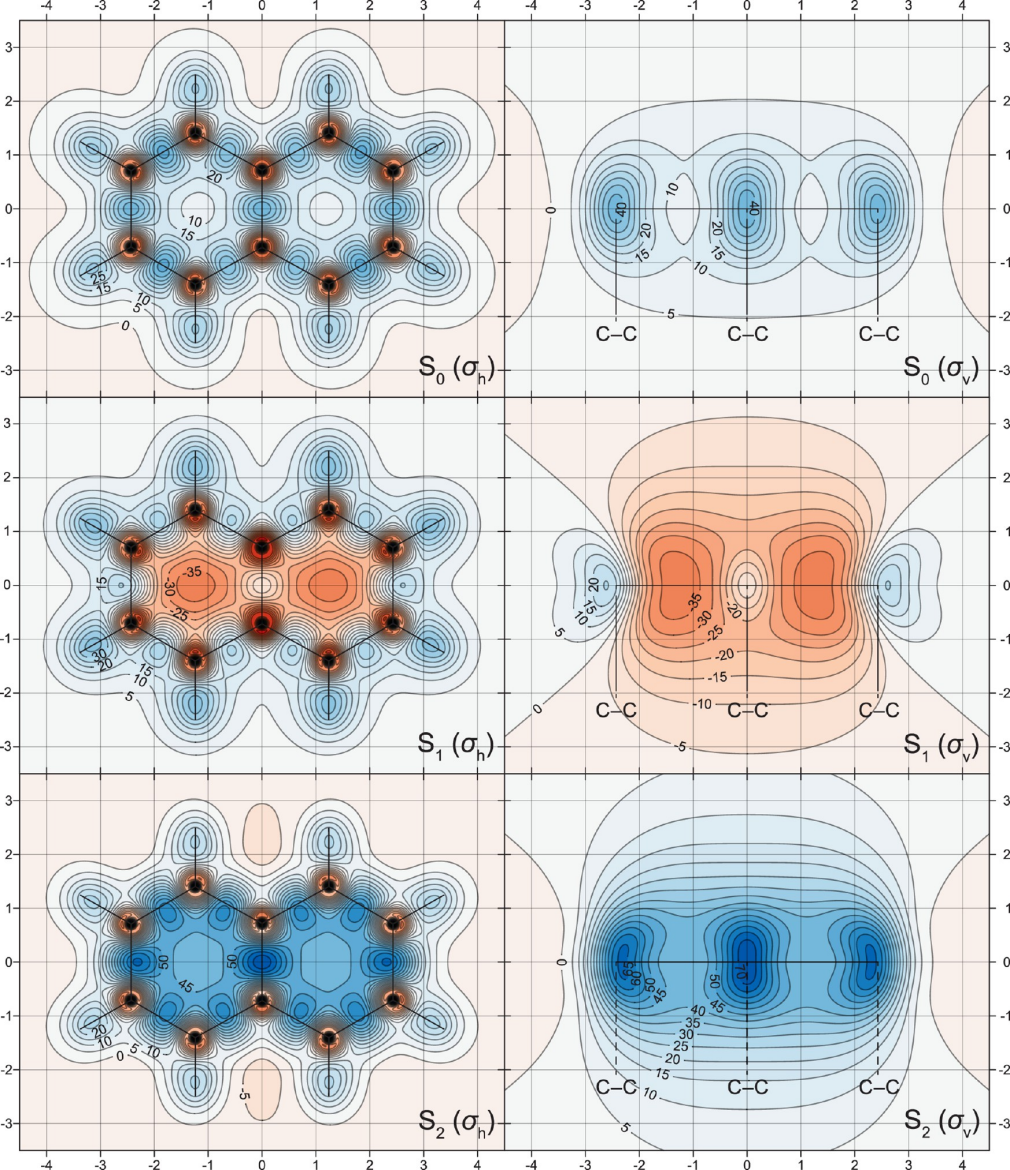
σ_iso_(**r**) contour plots for the S_0_, S_1_, and S_2_ states of naphthalene in the molecular (σ_h_) and longitudinal vertical (σ_v_) planes [CASSCF(10,10)-GIAO/6-311+G(d) results]. Contour levels at −75(5)75 ppm, orange (deshielded) to blue (shielded), axes in Å. Dashed lines show the positions of C–C bonds bisected by the σ_v_ plane.

**Figure 3:**
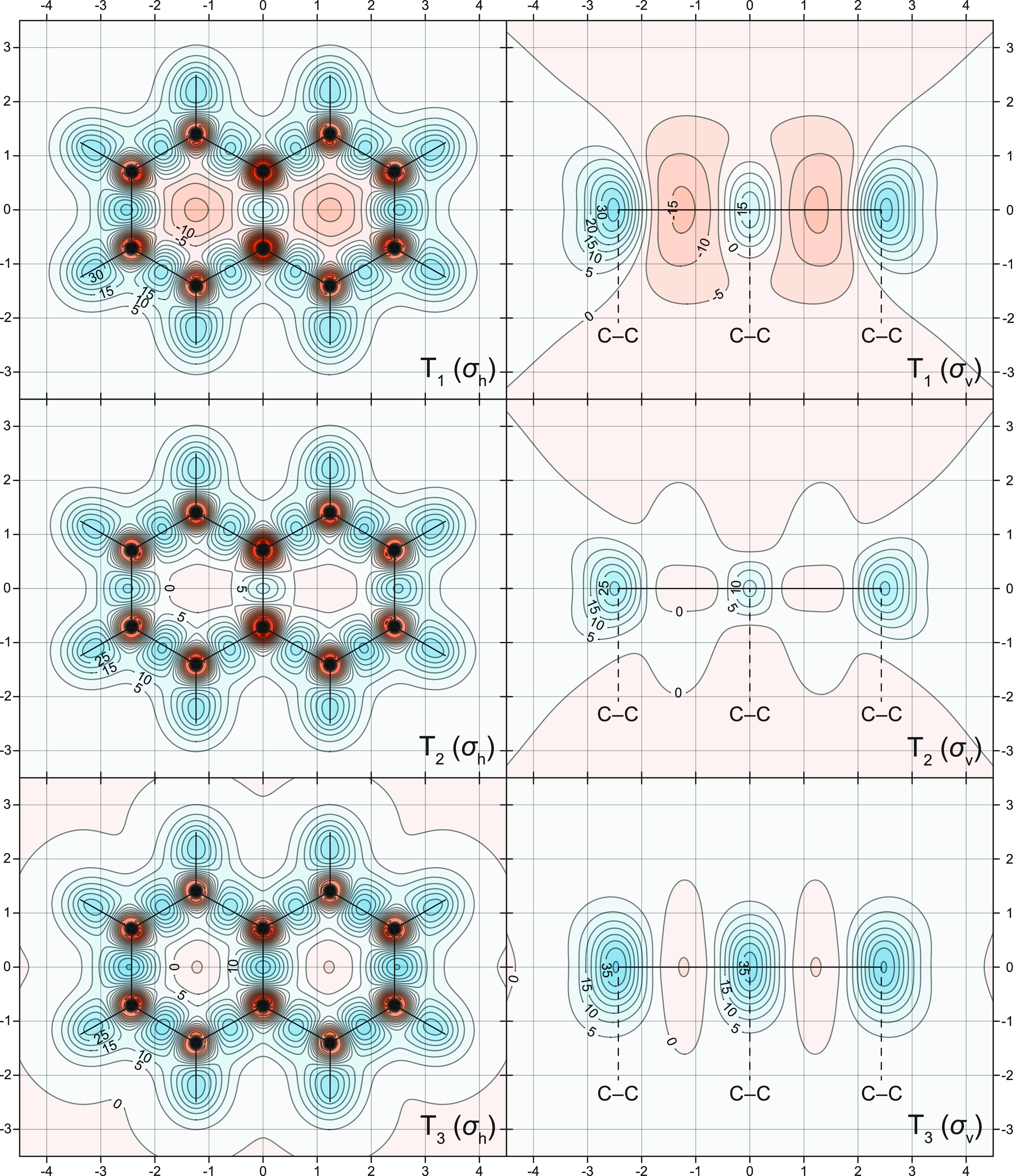
σ_iso_(**r**) contour plots for the T_1_, T_2_, and T_3_ states of naphthalene. Details as for Figure 2.

**Figure 4:**
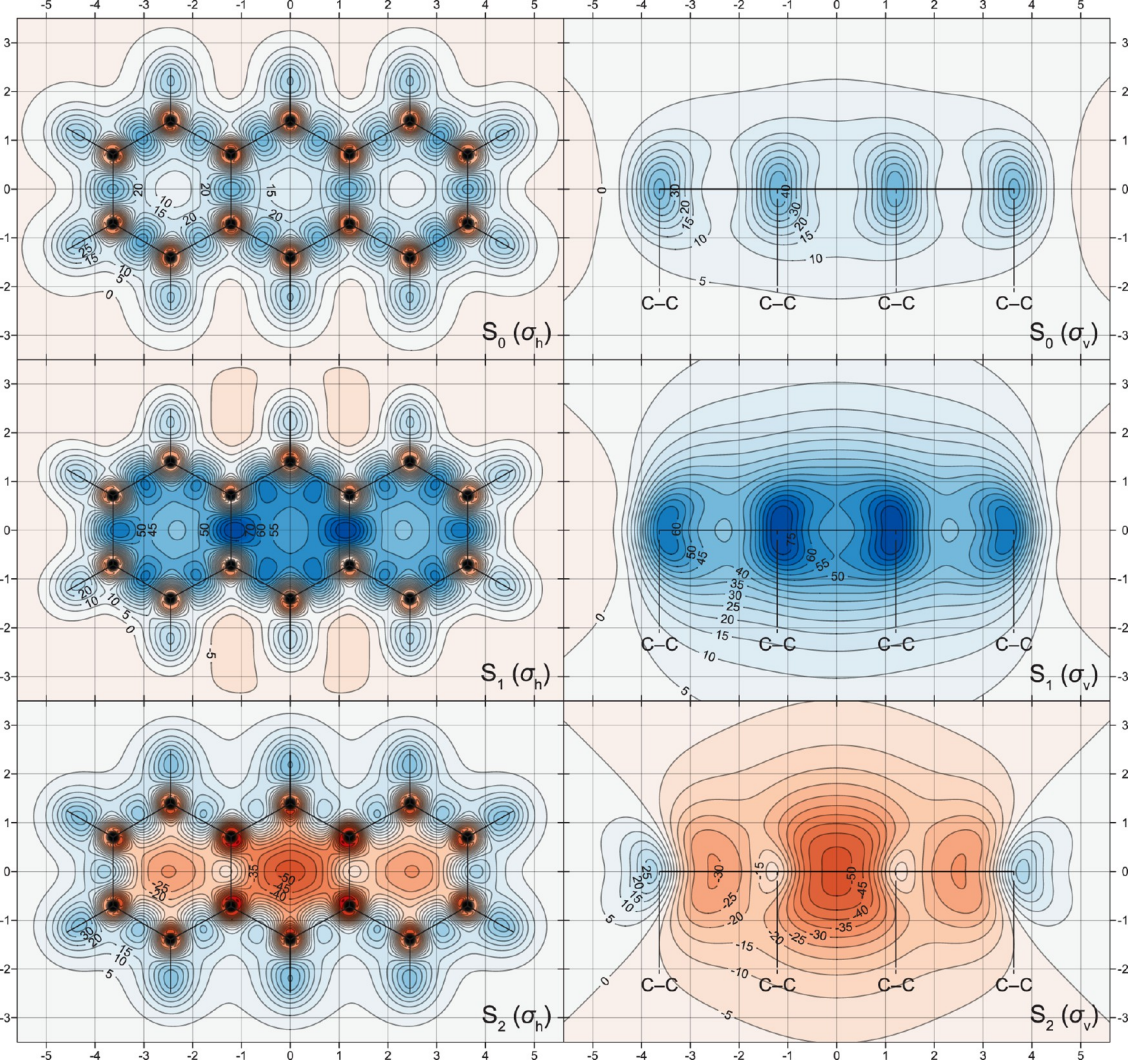
σ_iso_(**r**) contour plots for the S_0_, S_1_, and S_2_ states of anthracene in the molecular (σ_h_) and longitudinal vertical (σ_v_) planes [CASSCF(14,14)-GIAO/6-311G(d) results]. Other details as for Figure 2.

**Figure 5:**
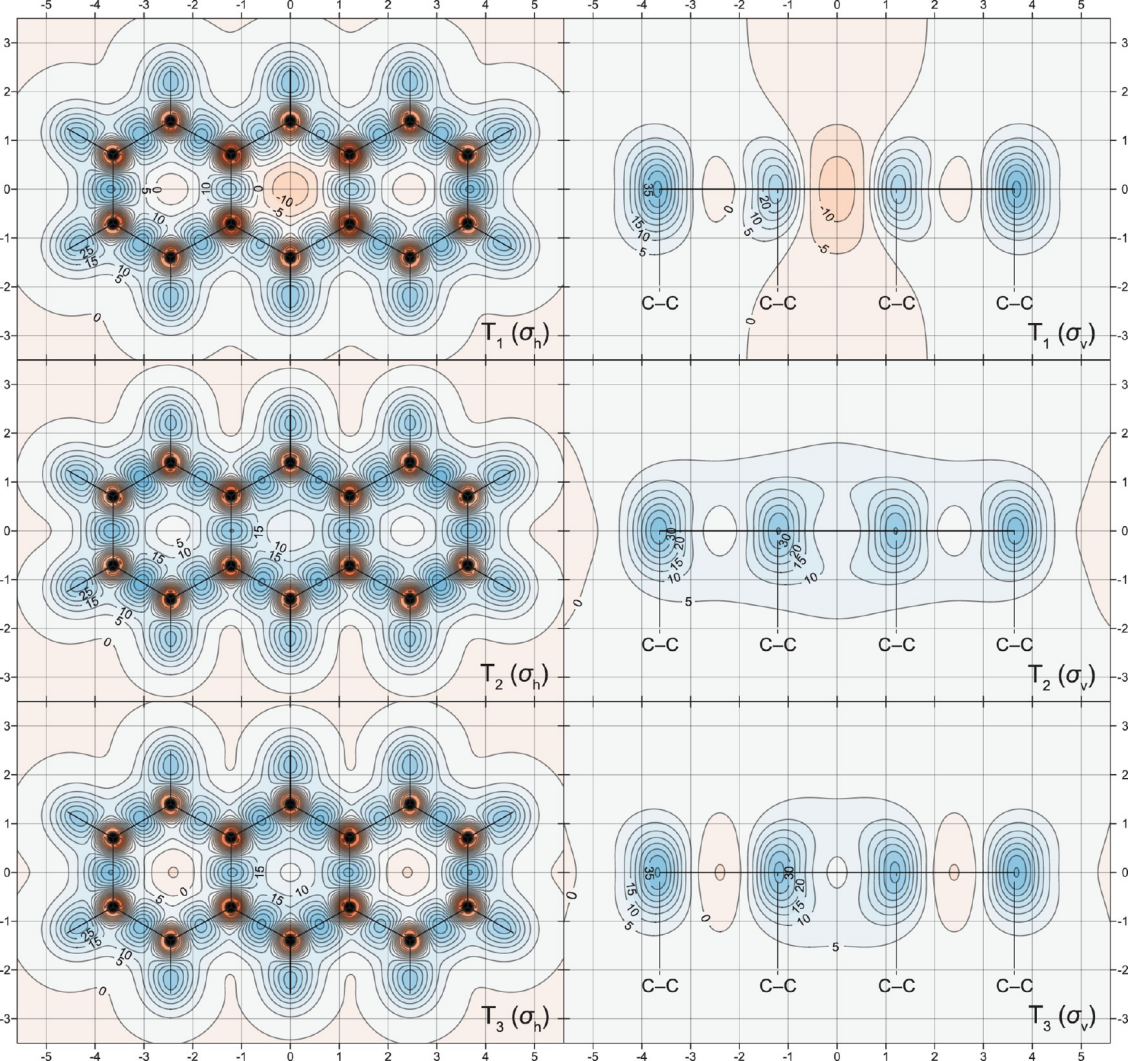
σ_iso_(**r**) contour plots for the T_1_, T_2_, and T_3_ states of anthracene. Details as for Figure 4.

The changes in off-nucleus isotropic shielding around naphthalene and anthracene in their S_0_, S_1_, S_2_, T_1_, T_2_, and T_3_ states are illustrated in Figures 1–5, through σ_iso_(**r**) isosurfaces (for naphthalene, Figure 1) and contour plots (for both molecules, Figures 2–5). The isovalues of σ_iso_(**r**) = +16 ppm in Figure 1 were chosen so as to display optimal levels of detail and to facilitate comparisons with the shielding isosurfaces for the S_0_, S_1_, S_2_, T_1_, and T_2_ states of benzene.^15^ Isosurfaces corresponding to other isovalues can be inspected using the Gaussian cube files provided in the Supporting Information. The σ_iso_(**r**) isosurfaces for the S_0_, S_1_, and S_2_ states of naphthalene look very similar to the outcomes that would be expected from fusing together the isosurfaces for the respective states of two benzene rings^15^ over the shared carbon–carbon bond. In the S_0_ state of naphthalene, the carbon rings are enclosed within a figure-of-eight shaped region of increased shielding, which suggests strong bonding interactions and aromatic stability. It is important to highlight here a difference between the pictures of chemical bonds in terms of electron density and off-nucleus shielding. The electron density along a chemical bond, when exposed to an external magnetic field, shields the bond, and this shielding persists even if the strength of the magnetic field approaches zero. The off-nucleus isotropic magnetic shielding usually increases and reaches a maximum near the midpoint of a bond, rendering most of the bond well-shielded, in contrast to electron density, which quickly decreases away from atoms. Hence, off-nucleus isotropic shielding plots tend to show higher levels of bond-specific details over the whole length of a chemical bond, which make the differences between bonds easier to visualize.^55^

The antiaromaticity of the S_1_ state of naphthalene follows from the presence of a large strongly deshielded central region, which, as portrayed by the σ_iso_(**r**) = −16 ppm isosurface, resembles two dumbbells, one inside each ring, interconnected over the shared carbon–carbon bond. This strongly deshielded region leads to a noticeable reduction of shielding over all peripheral carbon–carbon bonds, in comparison to the shielding picture in S_0_, and to displacement of this shielding toward the exterior of the carbon framework. The bond between the shared carbon atoms is completely deshielded (see also the S_1_ contour plots in Figure 2), and the σ_iso_(**r**) = +16 ppm isosurface features “holes” next to these carbon atoms. Similarly to the shielding pictures observed in the S_2_ states of benzene^15^ and cyclooctatetraene,^16^ in the S_2_ state of naphthalene, the interior of the carbon framework is well-shielded all over, which suggests a high level of aromaticity. The more shielded regions close to peripheral carbon–carbon bonds in S_2_ are displaced toward the interiors of two rings; higher shielding is observed next to the bonds between carbon atoms in β positions and over the bond between the shared carbon atoms (see the S_2_ contour plots in Figure 2).

In contrast to the similar levels of antiaromaticity observed in the S_1_ and T_1_ states of benzene,^15^ and of aromaticity observed in the S_1_ and T_1_ states of cyclobutadiene^15^ and cyclooctatetraene,^16^ shielding around the T_1_ state of naphthalene indicates that this state is significantly less antiaromatic than the S_1_ state. Instead of the large strongly deshielded central region observed in S_1_, all that remains of the σ_iso_(**r**) = −16 ppm isosurface within the ring interiors in T_1_ are two small deshielded “blobs” near the ring centers (Figure 1); the contour plots in Figure 3 show that the extent of deshielding within, above and below the rings in T_1_, is nonnegligible but still significantly less pronounced than that in S_1_ (Figure 2). While the visual differences between the S_1_ and T_1_ σ_iso_(**r**) = +16 ppm isosurfaces in Figure 1 are relatively minor, the contour plots in the σ_h_ plane (Figures 2 and 3) show that the shielding over the peripheral carbon–carbon bonds in T_1_ is higher and less displaced toward the exterior of the carbon framework than that in S_1_; in T_1_, the bond between the shared carbon atoms is no longer completely deshielded as the corresponding bond in S_1_. The shielding pictures around the T_2_ and T_3_ states of naphthalene suggest that both states are less antiaromatic than T_1_. The differences between the behaviors of σ_iso_(**r**) in the T_1_, T_2_, and T_3_ states are easier to observe in the respective contour plots in Figure 3. The deshielded regions within, above and below the rings in T_2_ and T_3_, are noticeably smaller and of lower intensities than their counterparts in T_1_; the carbon framework in T_2_ is slightly less well-shielded than that in T_1_, whereas all carbon–carbon bonds in T_3_ are relatively well-shielded (but still much less so than the carbon–carbon bonds in S_0_). These observations suggest that it would be appropriate to classify the T_2_ and T_3_ states of naphthalene as weakly antiaromatic and nonaromatic, respectively.

The most interesting observation following from the σ_iso_(**r**) contour plots for the S_0_, S_1_, and S_2_ states of anthracene (Figure 4) is that the S_1_ state is no longer antiaromatic; the strongly antiaromatic state is now S_2_, with an off-nucleus shielding distribution very similar to those in the S_1_ states of naphthalene and benzene; the off-nucleus shielding distribution in the S_1_ state in anthracene closely resembles those in the aromatic S_2_ states in naphthalene and benzene (see Figure 2 and ref (15)). This observation strongly suggests that, throughout the [*n*]acene series, the 1^1^B_3u_ (^1^L_b_) state, S_1_ for *n* = 1, 2 and S_2_ for *n* ≥ 3, which is dominated by the HOMO → LUMO excitation, will be antiaromatic, while the 1^1^B_2u_ (^1^L_a_) state, S_2_ for *n* = 1, 2 and S_1_ for *n* ≥ 3, which is dominated by the HOMO −1 → LUMO and HOMO → LUMO +1 excitations, will be aromatic.

At first glance, the σ_iso_(**r**) contour plots for the S_0_, S_1_, and S_2_ states of anthracene (Figure 4) look like extensions, by one ring each, of the σ_iso_(**r**) contour plots for the S_0_, S_2_, and S_1_ states of naphthalene (Figure 3). Closer scrutiny reveals that both local aromaticity and local antiaromaticity are more pronounced in the central ring: according to the σ_iso_(**r**) contour plots in Figure 4, in the S_0_ state, the central ring is only slightly more aromatic than the outer rings, whereas this ring is noticeably more aromatic or antiaromatic than the outer rings in the S_1_ and S_2_ states, respectively. Comparing anthracene to naphthalene, in S_0_, the central ring in C_14_H_10_ is slightly more shielded than the rings in C_10_H_8_, and the outer rings are shielded very similarly to the rings in C_10_H_8_; in S_1_, the central ring in C_14_H_10_ is noticeably more shielded, and the outer rings are noticeably less shielded than the rings in the S_2_ state of C_10_H_8_; in S_2_, the central ring in C_14_H_10_ is considerably more deshielded, and the outer rings are noticeably less deshielded than the rings in the S_1_ state of C_10_H_8_. The shielding over the carbon–carbon bonds in the S_0_, S_1_, and S_2_ states of anthracene is very similar to the shielding over the corresponding carbon–carbon bonds in the S_0_, S_2_, and S_1_ states of naphthalene.

The σ_iso_(**r**) contour plots for the T_1_ state of anthracene (Figure 5) show that the interiors of the central and outer rings are considerably less deshielded than the interiors of the corresponding rings in the S_1_ state (Figure 4), which is an indication of much lower levels of local antiaromaticity. In fact, the very weak deshielding of the interiors of the outer rings in the T_1_ state suggests that these rings should be considered as locally nonaromatic; even the more pronounced deshielding of the central ring is insufficient to cause significant displacements of the shielded regions over the carbon–carbon bonds from this ring toward its exterior. The comparison between the σ_iso_(**r**) contour plots for the S_0_ and T_2_ states of anthracene (Figures 4 and 5) reveals a degree of similarity, which is sufficient to allow classification of the T_2_ state as aromatic, albeit less so than the S_0_ state. In the T_3_ state of anthracene (Figure 5), the interiors of the central and outer ring are relatively weakly shielded and deshielded, respectively, which suggests that this state should be considered as nonaromatic.

**Figure 6:**
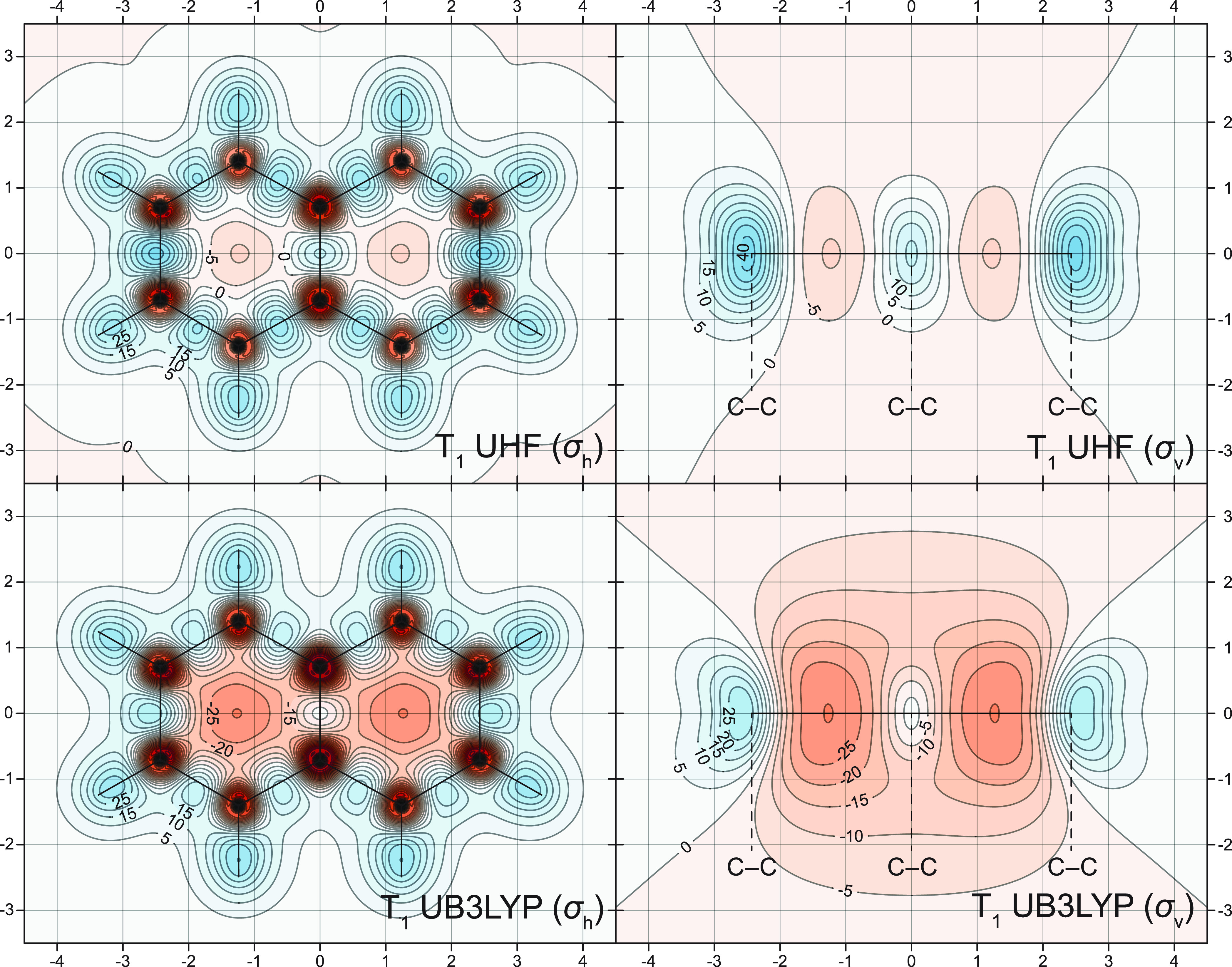
UHF-GIAO and UB3LYP-GIAO σ_iso_(**r**) contour plots for the T_1_ state of naphthalene in the molecular (σ_h_) and longitudinal vertical (σ_v_) planes [6-311+G(d) basis set]. Other details as for Figure 2.

**Figure 7:**
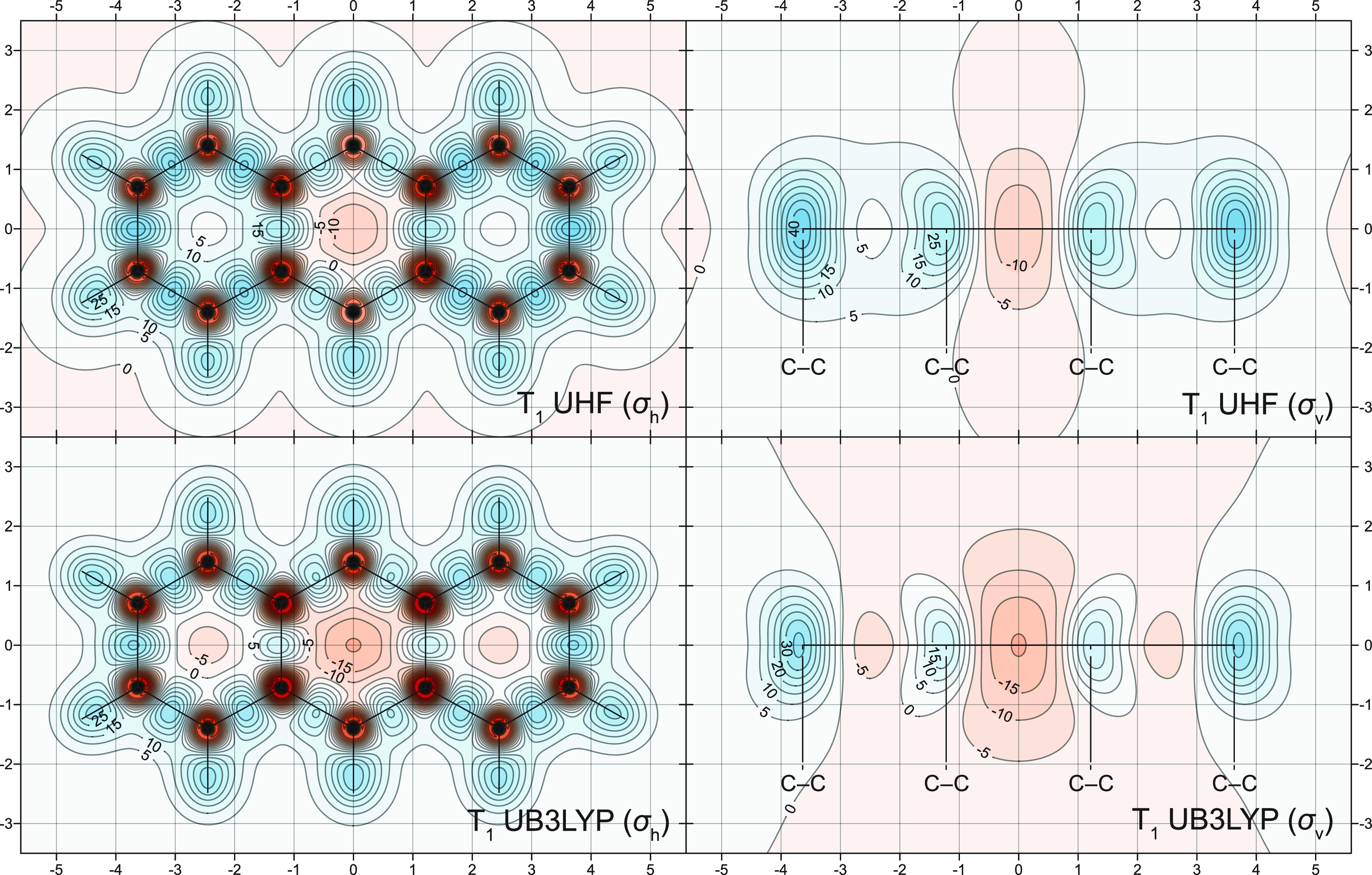
UHF-GIAO and UB3LYP-GIAO σ_iso_(**r**) contour plots for the T_1_ state of anthracene in the molecular (σ_h_) and longitudinal vertical (σ_v_) planes [6-311G(d) basis set]. Other details as for Figure 2.

Contour plots illustrating how the UHF-GIAO and UB3LYP-GIAO methods account for the variations of the off-nucleus isotropic shielding around the T_1_ states of naphthalene and anthracene are included in Figures 6 and 7. The comparison between the CASSCF(10,10)-GIAO, UHF-GIAO, and UB3LYP-GIAO σ_iso_(**r**) contour plots for the T_1_ state of naphthalene (Figures 3 and 6) shows that the antiaromaticity of this state is significantly overestimated at the UB3LYP-GIAO level and somewhat underestimated at the UHF-GIAO level. It should be noted that, even as overestimated by the UB3LYP-GIAO method, the antiaromaticity of the T_1_ state of naphthalene is not so pronounced as that of the S_1_ state (compare Figures 2 and 6). The differences between the CASSCF(14,14)-GIAO, UHF-GIAO, and UB3LYP-GIAO σ_iso_(**r**) contour plots for the T_1_ state of anthracene (Figures 5 and 7) are relatively smaller than those between their counterparts for naphthalene. The UB3LYP-GIAO method still overestimates the antiaromaticity of this state, but less so than in the case of naphthalene. UHF-GIAO and CASSCF(14,14)-GIAO are in reasonable agreement about the level of antiaromaticity of the central ring, but UHF-GIAO describes the outer rings as weakly aromatic rather than nonaromatic. These observations suggest that, for larger [*n*]acenes and, very likely, for larger PAHs in general, the agreement between off-nucleus isotropic shieldings for the T_1_ state calculated at the state-optimized π space CASSCF-GIAO, UHF-GIAO, and UB3LYP-GIAO levels can be expected to improve. On the other hand, the differences between the levels of antiaromaticity in the T_1_ state and in the strongly antiaromatic low-lying singlet-state (S_1_ for naphthalene and S_2_ for anthracene and larger [*n*]acenes) can be expected to remain substantial. Other descriptions of local antiaromaticity in the T_1_ states of PAHs obtained using UB3LYP, such as those of acenes and phenacenes reported in ref (56), may also overestimate its levels, and it would be advisable to include in such studies, if the size of the PAH makes π space CASSCF calculations infeasible, comparisons to analogous descriptions obtained using UHF in which the levels of local antiaromaticity are more likely to be underestimated.

The carbon atoms in all electronic states of naphthalene and anthracene studied in this paper are surrounded by small ovoid deshielded regions inside, which σ_iso_(**r**) becomes negative. These regions are more noticeable in the contour plots in Figures 2–7. Similar deshielded “halos” around sp^2^ and sp hybridized carbon atoms and other sp^2^ hybridized first main row atoms have been observed previously in conjugated rings,^15,57−59^ as well as in open-chain conjugated molecules such as ethene, ethyne, and *s*-trans-1,3-butadiene.^55,60^ These “halos” have been attributed to a specific type of π electron behavior that is characteristic of some sp^2^ and sp hybridized first main row atoms and that is different from traditional ring currents.^15^ The sizes and intensities of the deshieded “halos” surrounding the carbon atoms in the different electronic states of naphthalene and anthracene are quite similar, with the exception of those observed in the strongly antiaromatic singlet electronic states (S_1_ in naphthalene and S_2_ in anthracene), in which the deshielding within the “halos” is more intensive, especially around shared carbon atoms.

An alternative way of investigating the aromaticities of the electronic states of naphthalene and anthracene is to examine, instead of σ_iso_(**r**), the behavior of the *zz* (out-of-plane) component of the off-nucleus shielding tensor, σ_*zz*_(**r**). This approach can be viewed as a generalization of the calculations of single-point NICS_*zz*_(0) and NICS_zz_(1) values and linear NICS-*XY*-scans^61^ and allows more straightforward comparisons to ring current plots. As a rule, the σ_*zz*_(**r**) contour plots look like accentuated versions of the respective σ_iso_(**r**) contour plots and their analysis leads to very much the same conclusions. According to our experience based on comparisons between σ_iso_(**r**) and σ_*zz*_(**r**) contour plots for the electronic ground states of various PAHs,^27,62^ it is simpler and more informative to work with the σ_iso_(**r**) plots, which retain relation to bonding, rather than with the σ_*zz*_(**r**) plots, which reflect some of the features of ring currents but may prove more complicated to analyze. As demonstrated by the work of other authors,^63−65^ σ_*zz*_(**r**) and related plots represent a viable alternative to σ_iso_(**r**) plots when studying planar conjugated molecules. While it is possible to choose between σ_iso_(**r**) and σ_*zz*_(**r**) contour plots when describing planar molecules, the description of aromaticity in nonplanar conjugated molecules requires the use of σ_iso_(**r**) isosurfaces.^66,67^

Clearly, the shielding isosurfaces and contour plots in Figures 1–7 contain much additional information in comparison to single-point NICS values. However, it is still interesting to investigate the extent to which different types of NICS can reproduce the aromaticity trends in the S_0_, S_1_, S_2_, T_1_, T_2_, and T_3_ states of naphthalene and anthracene. The NICS(0), NICS(1), NICS(0)_*zz*_, and NICS(1)_*zz*_ values for these states are shown in Table 2 together with the corresponding χ_iso_ and χ_*zz*_ values. The signs of the four NICS values for all six-membered rings in of the S_0_, S_1_, and S_2_ states of naphthalene and anthracene are, as expected from Figures 1, 2, and 4, either negative (for the aromatic S_0_ and S_2_ states of naphthalene, and S_0_ and S_1_ states of anthracene) or positive (for the antiaromatic S_1_ state of naphthalene and S_2_ state of anthracene); the magnitudes of the NICS values for the outer rings of anthracene are smaller than those for the central ring. All four NICS values for the T_1_ state of naphthalene are positive but significantly smaller than their counterparts for the S_1_ state, which confirms that, in naphthalene, the T_1_ state is antiaromatic but much less so than the S_1_ state. While the NICS data for the T_1_ state of anthracene unambiguously classify the central ring as antiaromatic, the only “antiaromatic” NICS value for the outer rings is that of NICS(0)_*zz*_; according to the other three NICS values, the outer rings in this state are nonaromatic. The NICS values for the T_2_ and T_3_ states of naphthalene and anthracene are a mixed bag. The T_2_ and T_3_ states of naphthalene come out as nonaromatic according to NICS(0) and NICS(1), antiaromatic according to NICS(0)_*zz*_, and weakly aromatic according to NICS(1)_*zz*_. The central ring of anthracene is aromatic in the T_2_ state and weakly aromatic in the T_3_ state according to the NICS(0), NICS(1), and NICS(1)_*zz*_, while the NICS(0)_*zz*_ values classify this ring as nonaromatic in T_2_ and weakly antiaromatic in T_3_. The outer rings of anthracene are weakly aromatic in the T_2_ state and nonaromatic or weakly antiaromatic in the T_3_ state according to the NICS(0), NICS(1), and NICS(1)_*zz*_ values; the NICS(0)_*zz*_ values suggest that these rings are antiaromatic in both of T_2_ and T_3_, and more so in T_3_. We observe that the NICS(0)_*zz*_ values in the T_2_ and T_3_ states of naphthalene and anthracene are often out of line with the respective NICS(0), NICS(1), and NICS(1)_*zz*_ values, which suggests that, for these states, NICS(0)_*zz*_ is not a reliable aromaticity criterion. While the NICS(0), NICS(1), and NICS(1)_*zz*_ values in the T_2_ and T_3_ states of naphthalene and anthracene are in agreement with the conclusions following from the examination of the shielding isosurfaces and contour plots in Figures 1, 3, and 5, it is certainly much easier to decide on the levels of aromaticity in these state by comparing the shielding variations in molecular space and, especially, around carbon–carbon bonds. The NICS values for the T_1_ states of naphthalene and anthracene calculated at the UHF-GIAO and UB3LYP-GIAO levels support the conclusions made while examining the shielding contour plots in Figures 6 and 7.

**Table 2:** NICS Values (in ppm) and χ_iso_ and χ_*zz*_ Values (in ppm cm^3^ mol^–1^) for the S_0_, S_1_, S_2_, T_1_, T_2_, and T_3_ States of Naphthalene and Anthracene^a^

molecule	method	state	NICS(0)	NICS(1)	NICS(0)_*zz*_	NICS(1)_*zz*_	χ_iso_	χ_*zz*_
C_10_H_8_	CASSCF	S_0_	–8.1	–9.8	–8.2	–25.4	–99.3	–176.2
		S_1_	37.7	28.3	127.8	87.9	9.9	147.2
		S_2_	–40.5	–37.4	–108.4	–109.5	–173.0	–415.6
		T_1_	16.1	10.3	64.1	34.8	–41.3	–4.8
		T_2_	3.7	–3.1	26.3	–5.8	–48.8	–29.3
		T_3_	5.2	1.2	30.9	–6.9	–67.8	–85.9
	UHF	T_1_	10.4	5.1	44.6	18.3	–52.2	–45.3
	UB3LYP	T_1_	30.1	21.4	102.0	67.0	–0.3	96.1
C_14_H_10_	CASSCF	S_0_	–10.7	–12.4	–12.7	–30.4	–137.9	–250.7
			–7.0	–9.3	–3.5	–21.9		
		S_1_	–52.3	–48.3	–138.2	–136.7	–263.1	–634.0
			–39.6	–36.6	–101.7	–103.9		
		S_2_	51.8	39.6	173.5	125.2	25.8	238.2
			30.4	22.1	108.2	71.9		
		T_1_	13.2	7.2	58.6	28.8	–85.7	–95.9
			2.5	–1.2	25.3	2.5		
		T_2_	–5.9	–9.2	1.2	–20.7	–117.6	–192.1
			–2.8	–6.2	8.9	–12.7		
		T_3_	–4.2	–6.7	7.3	–13.2	–100.4	–141.2
			5.2	0.7	32.3	7.5		
	UHF	T_1_	14.2	7.6	60.5	30.1	–95.4	–131.6
			–3.2	–6.2	7.7	–12.3		
	UB3LYP	T_1_	20.3	12.8	77.6	45.2	–59.2	–37.0
			7.3	2.3	36.5	12.5		

a Naphthalene: CASSCF(10,10)-GIAO, UHF-GIAO, and UB3LYP-GIAO results in the 6-311+G(d) basis; anthracene: CASSCF(14,14)-GIAO, UHF-GIAO, and UB3LYP-GIAO results in the 6-311G(d) basis. Anthracene NICS: central ring (top values), outer rings (bottom values).

The ranges of χ_iso_ and χ_*zz*_ values shown in Table 2 allow a classification of the low-lying electronic states of naphthalene and anthracene as aromatic, nonaromatic, and antiaromatic, which is in full agreement with the results of the analyses of the shielding isosurfaces, contour plots, and NICS values for these states. It is important to note that the χ_iso_ and χ_*zz*_ values characterize the aromaticity of the whole molecule rather than that of a single ring in a particular electronic state. As a consequence, the χ_iso_ and χ_*zz*_ values for naphthalene and anthracene should not be compared directly; division by two or three, respectively, will provide “per ring” values, which can be compared; however, these will be only average “per ring” values for anthracene and will not reflect the different levels of aromaticity of the central and outer rings.

The method dependence of magnetic properties associated with the “anthracene problem” is illustrated by the results reported in Table 3, which include ground-state NICS(0), NICS(1), NICS(0)_zz_, NICS(1)_*zz*_, χ_iso_, and χ_*zz*_ values for benzene, naphthalene, and anthracene calculated in the 6-311G(d) basis using full π space CASSCF-GIAO wavefunctions, CASSCF(6,6)-GIAO, CASSCF(10,10)-GIAO, and CASSCF(14,14)-GIAO, respectively, and the MP2-GIAO, CC2-GIAO, and CC3-GIAO methods.

**Table 3:** NICS Values (in ppm) and χ_iso_ and χ_*zz*_ Values (in ppm cm^3^ mol^–1^) for the Electronic Ground States of Benzene, Naphthalene, and Anthracene^a^

molecule	method	NICS(0)	NICS(1)	NICS(0)_*zz*_	NICS(1)_*zz*_	χ_iso_	χ_*zz*_
C_6_H_6_	CASSCF	–8.9	–10.6	–11.2	–27.1	–59.7	–99.5
	MP2	–8.6	–11.3	–14.4	–29.8	–58.8	–102.6
	CC2	–8.6	–11.2	–14.3	–29.6	–58.7	–101.9
	CC3	–8.5	–10.9	–13.2	–28.5	–61.2	–103.6
C_10_H_8_	CASSCF	–8.6	–10.5	–8.3	–25.5	–99.3	–176.0
	MP2	–9.1	–11.6	–13.4	–29.7	–99.7	–186.7
	CC2	–9.1	–11.6	–13.3	–29.5	–99.4	–185.6
	CC3	–9.1	–11.3	–12.7	–28.6	–119.3	–210.9
C_14_H_10_	CASSCF	–10.7	–12.4	–12.7	–30.4	–137.9	–250.7
		–7.0	–9.3	–3.5	–21.9		
		–8.2	–10.3	–6.5	–24.7		
	MP2	–11.8	–14.0	–19.4	–35.9	–140.3	–271.4
		–8.0	–10.9	–10.1	–27.3		
		–9.3	–11.9	–13.2	–30.2		
	CC2	–11.8	–14.0	–19.2	–35.6	–139.8	–269.9
		–8.0	–10.8	–10.0	–27.2		
		–9.3	–11.9	–13.1	–30.0		
	CC3	–11.8	–13.7	–19.0	–34.9	–202.2	–363.4
		–8.2	–10.6	–9.7	–26.4		
		–9.4	–11.6	–12.8	–29.3		

a CASSCF-GIAO, MP2-GIAO, CC2-GIAO, and CC3-GIAO results in the 6-311G(d) basis; CASSCF(6,6) for benzene, CASSCF(10,10) for naphthalene, and CASSCF(14,14) for anthracene. Anthracene NICS: central ring (top values), outer rings (middle values), and average (bottom values).

The ground-state CASSCF(6,6)-GIAO and CASSCF(10,10)-GIAO NICS(0), NICS(1), NICS(0)_*zz*_, NICS(1)_*zz*_, χ_iso_, and χ_*zz*_ values for benzene and naphthalene calculated in the 6-311G(d) basis show only minor differences from the respective results in 6-311+G(d) basis (see ref (8) and Table 2) and support the conclusion that, according to these NICS values and the χ_iso_ and χ_*zz*_ “per ring” values, each of the six-membered rings in naphthalene is less aromatic than benzene.^8^ It is less straightforward to distinguish between the aromaticities of the six-membered rings in benzene and naphthalene on the basis of the MP2-GIAO, CC2-GIAO, and CC3-GIAO results. The NICS(0) and NICS(1) values calculated at these levels of theory incorrectly assign slightly higher aromaticity to each of the naphthalene rings; the ordering of the NICS(0)_zz_ values is in agreement with the CASSCF-GIAO results, but the differences between benzene and naphthalene are smaller; of the NICS(1)_*zz*_ values, the MP2-GIAO and CC2-GIAO results show the correct ordering, but the CC3-GIAO results fail to do so; for all three approaches, the differences between the NICS(1)_*zz*_ values for the two molecules are very small, ca. ±0.1 ppm. The MP2-GIAO and CC2-GIAO χ_iso_ and χ_*zz*_ values for benzene and naphthalene are reasonably close to the respective CASSCF-GIAO results, and the CC3-GIAO χ_iso_ and χ_*zz*_ values for benzene are only slightly larger in magnitude. However, the magnitudes of the CC3-GIAO χ_iso_ and χ_*zz*_ values for naphthalene are substantially larger than those of the corresponding MP2-GIAO and CC2-GIAO results; the differences between the MP2-GIAO/CC2-GIAO and CC3-GIAO χ_iso_ and χ_*zz*_ magnitudes in anthracene are even larger. The large differences between the CC3-GIAO χ_iso_ and χ_*zz*_ values for naphthalene and anthracene and the results for these quantities obtained using all other methods suggest that the CC3-GIAO values are inaccurate, and we are not going to discuss them any further.

According to all NICS values reported in Table 3, the central ring in anthracene should be more aromatic than the outer rings. The differences between the NICS values for the two types of rings are similar for all four methods; the NICS values calculated at the MP2-GIAO, CC2-GIAO, and CC3-GIAO levels overestimate the aromaticities of both rings in comparison to their CASSCF(14,14)-GIAO counterparts. To compare the NICS predictions for the relative aromaticities of benzene, naphthalene, and anthracene, following Schleyer and co-workers,^21^ we provide average NICS values for anthracene (Table 3). All four types of average NICS values calculated at the CASSCF(14,14)-GIAO and CASSCF(10,10)-GIAO levels for anthracene and naphthalene, respectively, indicate that the average aromaticity of a six-membered ring in anthracene is lower than that of a six-membered ring in naphthalene. Thus, NICS values calculated with full π space CASSCF-GIAO wavefunctions reproduce the correct ordering of benzene, naphthalene, and anthracene according to their average “per ring” aromaticity levels. At the MP2-GIAO and CC2-GIAO levels, the only anthracene average NICS value suggesting a decrease of “per ring” aromaticity in comparison to naphthalene is NICS(0)_*zz*_; at the CC2-GIAO level, all four anthracene average NICS values suggest “per ring” aromaticity higher than that in naphthalene. This shows that, in general, similarly to the DFT-IGLO approach employed in ref (21), PW91-IGLO/IGLO-III, average NICS values calculated with the MP2-GIAO, CC2-GIAO, and CC3-GIAO methods do not provide reliable criteria for comparing the “per ring” aromaticities of naphthalene and anthracene. On the other hand, the MP2-GIAO and CC2-GIAO “per ring” χ_iso_ and χ_*zz*_ values for anthracene are lower in magnitude than the corresponding values for naphthalene and correctly reflect the expected decrease in the “per ring” aromaticity.

## 4 Conclusions

Somewhat unexpectedly, in contrast to benzene, square cyclobutadiene, and regular octagonal cyclooctatetraene, in which the levels of antiaromaticity or aromaticity in the S_1_ and T_1_ states have been found to be very similar,^13−16^ the state-optimized full π space CASSCF-GIAO calculations on these states in naphthalene and anthracene show that, in both molecules, the first antiaromatic singlet excited state (S_1_ in naphthalene and S_2_ in anthracene) is significantly more antiaromatic than the T_1_ state. Therefore, it would be incorrect to assume that the close similarity between the aromatic properties of the S_1_ and T_1_ states of benzene, square cyclobutadiene, and regular octagonal cyclooctatetraene would be maintained in polyacenes and PAHs in general; the current results on naphthalene and anthracene strongly suggest otherwise. Two popular computationally inexpensive methods for investigating the magnetic properties of the T_1_ state in larger conjugated molecules, UB3LYP-GIAO and UHF-GIAO, are shown to exaggerate and downplay, respectively, the antiaromaticity of the T_1_ state in naphthalene, in comparison to full π space CASSCF-GIAO. However, the differences between the results obtained with the three methods for anthracene are less pronounced, which suggests that, for larger polyacenes and PAHs, the UB3LYP-GIAO and UHF-GIAO methods can be expected to produce off-nucleus isotropic shieldings similar to those that would be obtained from a full π space CASSCF-GIAO calculation. The isotropic shielding distributions surrounding the triplet states of anthracene (Figure 5) and the corresponding NICS values (Table 3) suggest that some low-lying triplet states of polyacenes and PAHs could involve combinations of weakly antiaromatic and aromatic six-membered rings.

While the T_1_, T_2_, T_3_ states of naphthalene and anthracene are found to exhibit varying levels of aromaticity, the differences between the isotropic shielding distributions in these states are much less pronounced than those observed between the S_0_, S_1_, and S_2_ states of these molecules: the moderately antiaromatic T_1_ state is followed by a weakly antiaromatic T_2_ and a nonaromatic T_3_ in naphthalene, and by a moderately aromatic T_2_ and a nonaromatic T_3_ in anthracene.

The CASSCF(14,14)-GIAO results for the magnetic properties of the electronic ground state of anthracene show that, even with inclusion of nondynamic electron correlation effects through the use of a full π space CASSCF wavefunction, the central ring remains more aromatic than the outer rings. However, in contrast to the MP2-GIAO, CC2-GIAO, and CC3-GIAO methods, which include dynamic electron correlation effects, the full π space CASSCF-GIAO results for the magnetic properties of the electronic ground states of benzene, naphthalene, and anthracene show correctly that the average “per ring” aromaticity decreases in the sequence benzene, naphthalene, anthracene.

In fact, attempts to obtain a convincing theoretical proof that the central ring in anthracene is less aromatic than the outer rings are very likely to fail, as can be demonstrated by using simple valence bond (VB) arguments. Anthracene has two pairs of equivalent Kekulé resonance structures; the structures from one of the pairs involve two fused fully conjugated six-membered rings, one of which is the central ring, and the structures from the other pair involve one fully conjugated six-membered ring, which is one of the outer rings. These four Kekulé resonance structures will provide the main contributions to a π space valence bond (VB) wavefunction for anthracene, or to a VB representation of the respective CASSCF wavefunction obtained using CASVB.^68^ We can assume that if the weight of a structure involving two fused fully conjugated six-membered rings in a VB wavefunction is *x*, then the weight of a structure involving a single six-membered fully conjugated ring will be approximately *x*/2. With this assumption, the combined weight of the structures in which the central ring is fully conjugated becomes *x* + *x* = 2*x*, and the combined weight of the structures in which one chosen outer ring is fully conjugated becomes *x* + *x*/2 = 1.5*x*. According to the results of VB self-consistent field (VBSCF) calculations on anthracene,^69^ the ratio between the weights of the two types of Kekulé resonance structures is larger than 2, which makes the structures in which the central ring is fully conjugated even more predominant. As a consequence, the central ring in anthracene involves more resonance and can be thought to be more aromatic than the outer rings.

## Supporting Information Available

Details of the contents of the ZIP archive (PDF)
Archive of Gaussian cube files with isotropic shielding values for the S_0_, S_1_, S_2_, T_1_, T_2_, and T_3_ states of naphthalene (ZIP)
