# Supplementary material for: Excited-State Aromaticity Reversals in Naphthalene and Anthracene
Source: J Phys Chem A. 2023 Apr 3;127(14):3148–62. doi: 10.1021/acs.jpca.3c00485 (PMC10108368; doi:10.1021/acs.jpca.3c00485)
Supplement: Supplementary file 1 — jp3c00485_si_001.pdf [file jp3c00485_si_001.pdf]

Supporting Information

Excited State Aromaticity Reversals in Naphthalene and Anthracene

Peter B. Karadakov* and Muntadar A. H. Al-Yassiri

Department of Chemistry, University of York, Heslington, York YO10 5DD, U.K.

*E-mail: peter.karadakov@york.ac.uk

A zip archive of Gaussian cube files with isotropic shielding values for the S_0, S_1, S_2, T_1, T_2 and T_3 electronic states of naphthalene and Figures 2−7 in scalable vector graphics format (.svg) is available as a separate download. Details about the files included in the zip archive are provided in the table below.

naphthalene-isotropic-shielding-s0.cube	Gaussian cube file with isotropic shielding values for S_0 in naphthalene.
naphthalene-isotropic-shielding-s1.cube	Gaussian cube file with isotropic shielding values for S_1 in naphthalene.
naphthalene-isotropic-shielding-s2.cube	Gaussian cube file with isotropic shielding values for S_2 in naphthalene.
naphthalene-isotropic-shielding-t1.cube	Gaussian cube file with isotropic shielding values for T_1 in naphthalene.
naphthalene-isotropic-shielding-t2.cube	Gaussian cube file with isotropic shielding values for T_2 in naphthalene.
naphthalene-isotropic-shielding-t3.cube	Gaussian cube file with isotropic shielding values for T_3 in naphthalene.
ESARs-in-Naphthalene-and-Anthracene-Fig-2.svg	Scalable Vector Graphics file for Figure 2
ESARs-in-Naphthalene-and-Anthracene-Fig-3.svg	Scalable Vector Graphics file for Figure 3
ESARs-in-Naphthalene-and-Anthracene-Fig-4.svg	Scalable Vector Graphics file for Figure 4
ESARs-in-Naphthalene-and-Anthracene-Fig-5.svg	Scalable Vector Graphics file for Figure 5
ESARs-in-Naphthalene-and-Anthracene-Fig-6.svg	Scalable Vector Graphics file for Figure 6
ESARs-in-Naphthalene-and-Anthracene-Fig-7.svg	Scalable Vector Graphics file for Figure 7

The Gaussian cube files from the zip archive were used to prepare Figure 1 in the paper. These files allow examination of the isotropic shielding distributions in the S_0, S_1, S_2, T_1, T_2 and T_3 electronic states of naphthalene in greater detail by means of a suitable viewing program, for example GaussView (https://gaussian.com/gaussview6/, accessed on Jan 19, 2023) or Visual Molecular Dynamics (VMD, https://www.ks.uiuc.edu/Research/vmd/, accessed on Jan 19, 2023).

The scalable graphics files for Figures 2−7 allow detailed examination of the contour plots in these figures and can be viewed using the current versions of most web browsers.
